# Are Endodontic Solvents Cytotoxic? An In Vitro Study on Human Periodontal Ligament Stem Cells

**DOI:** 10.3390/pharmaceutics14112415

**Published:** 2022-11-08

**Authors:** José Luis Sanz, Sergio López-García, Leopoldo Forner, Francisco Javier Rodríguez-Lozano, David García-Bernal, Sonia Sánchez-Bautista, Clara Puig-Herreros, Vicent Rosell-Clari, Ricardo E. Oñate-Sánchez

**Affiliations:** 1 Department of Stomatology, Faculty of Medicine and Dentistry, Universitat de València, 46010 Valencia, Spain; 2 Department of Dermatology, Stomatology, Radiology and Physical Medicine, Morales Meseguer Hospital, Faculty of Medicine, University of Murcia, 30008 Murcia, Spain; 3 Department of Health Sciences, Catholic University San Antonio of Murcia, 30107 Murcia, Spain; 4 Department of Basic Psychology, Speech Therapy University Clinic, Universitat de València, 46010 Valencia, Spain

**Keywords:** endodontic solvents, cytotoxicity, eucalyptol, chloroform, Endosolv

## Abstract

The aim of this study was to assess the influence of eucalyptol, chloroform, and Endosolv on the proliferative capability, cell viability, and migration rates of human periodontal ligament stem cells (hPDLSCs) in vitro. Solvent eluates were formulated following ISO 10993-5 guidelines, and 1%, 0.25%, and 0.1% dilutions were prepared. The HPDLSCs were isolated from the extracted third molars of healthy donors. The following parameters were assessed: cell viability via trypan blue and IC50 assays, cell migration via horizontal wound healing assay, cell morphology via cell cytoskeleton staining (phalloidin labeling), and cell oxidative stress via reactive oxygen species assay. The data were analyzed using one-way ANOVA and Tukey’s posthoc tests, and their significance was established at *p* < 0.05. Chloroform and eucalyptol exhibited significantly higher cytotoxicity on the hPDLSCs in vitro compared to the control group, as shown by the cell viability, migration, morphology, and reactive oxygen species release assays. Alternatively, Endosolv showed adequate cytotoxicity levels comparable to those of the control group. The cytotoxicity of the tested endodontic solvents increased in a dose-dependent manner. The results from the present study highlight the cytotoxicity of chloroform and eucalyptol. Thus, their limited and cautious use is recommended, avoiding solvent extrusion.

## 1. Introduction

During endodontic retreatment, a series of techniques and tools are often used to remove the previous root canal filling materials, such as rotary files (reciprocating or continuous rotation), manual files, ultrasonic instruments, and/or chemical solvents [1,2]. To date, various solvents have been analyzed for their efficacy in removing gutta-percha and root canal sealers from the inside of root canals. Their ability to remove endodontic fillers has been tested in different clinical situations, especially when the filling material is well-adapted and resistant to instrument penetration or, most critically, in pronounced root curvatures with a risk of root perforation [3]. However, evidence on the toxicity of endodontic solvents remains limited. This is crucial since these materials can be extruded through the apex and/or secondary foramina into the periapical tissues and could result in detrimental effects such as inflammation, irritation, cytotoxicity, and interference with the healing process [4,5].

The ideal solvent should be noncarcinogenic and nontoxic to the adjacent tissues, deliver efficient gutta-percha softening and/or sealer removal, remain functional for an adequate time, and be cost-effective. Several types of endodontic solvents are available, but none meet the aforementioned requirements [3]. Classically, chloroform was the solvent of choice due to its effectiveness in the dissolution of most root canal-filling materials. However, concerns about its carcinogenicity led to the search for alternative solvents [6]. Essential oils, such as eucalyptol or tetrachloroethylene, were proposed as biocompatible alternatives, although they have been considered less effective than chloroform for the removal of endodontic fillers [7]. Additionally, available evidence on their cellular response is limited [5].

Among the cellular components from periapical tissues, human periodontal ligament stem cells (hPDLSCs) stand out as a relevant cell population [8]. These cells are a subgroup of dental stem cells (DSCs), which possess a mesenchymal stem cell-like phenotype and multilineage differentiation potential [9]. hPDLSCs play a crucial role in the neoformation of periodontal tissues and the healing of existing periapical lesions. Consequently, the extrusion of solvents with inadequate biological properties may hinder the repair process and healing of periapical lesions [10].

Laboratory cell assays are used as a preliminary assessment of cytocompatibility since they are straightforward, fast, and standardized procedures that reflect the physiological status of living tissues [11]. In such assays, primary and immortalized cell lines are used to evaluate cellular response. Although human primary cell culture isolation and passages are complex and sensitive, they are preferred in cytotoxicity studies because they can develop an experimental environment that mimics physiological conditions and better reflects in vivo behavior [12,13].

Based on limited previous evidence on various cell lineages [14,15], we hypothesized that endodontic solvents might inhibit critical cellular functions, such as metabolic activity, induction of cell death, cell proliferation, and cell migration. Accordingly, the aim of the present laboratory study was to assess the physiological effects of chloroform, eucalyptol, and Endosolv on the proliferative capability, cell viability, and migration rates of hPDLSCs. The null hypothesis was that there were no significant differences among the different solvents regarding their cytotoxicity on hPDLSCs.

## 2. Materials and Methods

### 2.1. Preparation of Solvent Eluates

The studied materials were Eucalyptol (DentaFlux, Madrid, Spain), Endosolv (Septodont, Saint-Maur-des-Fossés, France), and chloroform (Fisher Scientific UK Ltd., Loughborough, UK). The composition of the studied materials is presented in Table 1. Material eluates were formulated following ISO 10993-5 guidelines. In order to formulate a 10% concentration, 1 mL of each solvent was blended with 9 mL of Dulbecco’s Modified Eagle Medium (DMEM) (Gibco, Thermo Fisher Scientific, Carlsbad, CA, USA) and filtered through a 0.22-µm syringe filter. Finally, eluates were diluted with DMEM to produce different dilutions (1%, 0.25%, and 0.1%).

### 2.2. Isolation and Culture of hPDLSCs

The protocol for cell extraction was previously approved by the Human Research Ethics Committee at the University of Murcia (ref. 2199/2018). Human periodontal tissues were obtained with written informed consent from 18–25-year-old healthy donors (*n* = 10), scheduled for the extraction of impacted third molars for orthodontic or surgical reasons. Extracted molars were collected antiseptically and placed into a physiological solution supplemented with antibiotics (1% penicillin/streptomycin (Gibco, Waltham, MA, USA)) to prevent contamination. Samples were delivered to a laboratory in the Biomedical Research Institute of Murcia (IMIB) within two hours. The molar sample size was based on a previous study with similar methodology [16]. After cleaning the tooth surface, periodontal tissues were obtained from the surface of the root’s middle and apical thirds. Next, periodontal tissues were sliced into smaller portions and digested with Collagenase type I solution (3 mg/mL; Gibco, USA) for 1 h at 37 °C. Lastly, the periodontal cells were placed in a T-25 cm^2^ cell culture flask with DMEM (Gibco) supplemented with 15% fetal bovine serum (FBS; Gibco) and 1% penicillin/streptomycin (Gibco) and transferred into an incubator.

The hPDLSCs used in the present study were characterized in a previous study by our research group [17], following the International Society of Cellular Therapy (ISCT) guidelines [18] to confirm their mesenchymal nature and trilineage differentiation potential. For the subsequent assays, cells from passages 2–4 were used, as performed in similar previous studies [19].

### 2.3. Trypan Blue Assay

The cells were exposed to different solvent concentrations and placed into contact with 2% trypan blue at a 1:10 dilution. Thereafter, the samples were transported into the Countess™ Automated Cell Counter (Invitrogen, MA, USA), and the viable and dead cells were counted in the quadrants of the four outer chambers. Lastly, the percentage of dead and viable cells after exposure to the tested solvents was determined. A basal measurement (0 h) was performed, and another measurement was performed after 24 h of culture.

### 2.4. IC50 Assay

A total of 3 × 10^3^ cells were seeded on 96-well plates with culture medium and stored for 24 h at 37 °C, with 5% CO_2_, and at 95% humidity. The culture medium was then removed, and PBS with the tested solvents dilutions (10%, 5%, 2.5%, 2%, 1.5%, 1%, 0.5%, 0.25%, 0.2%, 0.15%, 0.1%) was added for 2, 10, or 20 min. Samples were then rinsed with PBS, and an MTT reagent (Sigma Aldrich, St. Louis, MO, USA) was added to assess cell metabolic activity, following the manufacturer’s instructions. Plates were covered and kept in dark conditions for 4 h. When a purple precipitate was detectable, Dimethylsulfoxide (DMSO) (Sigma-Aldrich) was added to each well (100 μL/well) to solubilize the formazan crystals produced by viable cells after reducing the MTT reagent. Lastly, light absorbance per well was recorded by means of a microplate reader (ELx800; Bio-Tek Instruments) at 570 nm wavelength.

The MTT assay was performed for each of the conditions (2, 10, or 20 min of treatment with the different dilutions of the solvents) immediately after the treatment period (0 h, basal measurement) or after 24 h of culture.

In order to assess the cytotoxicity of the solvents, the doses of the studied solvents that could decrease cell viability by 50% after 0 and 24 h (half maximal inhibitory concentration (IC50)) were analyzed graphically by plotting the percentage of metabolic activity on the *y*-axis and the concentration percentage of each solvent on the *x*-axis; as performed in a previous study [20]. IC50 values were analyzed by nonlinear regression using GraphPad Prism software version 8.1.0 (GraphPad Software Inc, San Diego, CA, USA).

### 2.5. Wound Healing Assay

A horizontal wound healing assay was carried out to assess the migration ability of the hPDLSCs in response to the solvent eluates. The hPDLSCs were seeded onto 6-well plates (2 × 10^5^ cells per well; *n* = 3 for each solvent and control) and left to proliferate until cell confluency was observed. Then, a superficial horizontal scratch wound was made on each cell monolayer using a 200 μL sterilized pipette tip, and each well was rinsed three times to remove any residual cell debris. hPDLSC migration was observed via an optical microscope (Olympus, Japan), and images were captured at 24, 48, and 72 h post wounding for each solvent and control group. ImageJ software v1.48 (National Institutes of Health, Bethesda, MD, USA) was used to measure the percentage of open wound area at each time point relative to the same wound area at 0 h in the same well.

### 2.6. Cell Cytoskeleton Staining

Cell cytoskeletons were stained using fluorescent-phalloidin labeling to observe macrophage morphological changes, as performed by a previous study [21]. Briefly, 3 × 10^4^ cells were seeded on glass coverslips, allowed to adhere and spread, and cultured in a complete growth medium alone (control) or in a complete growth medium containing the different solvent eluates at 1%, 0.25%, and 0.1% concentrations for 24 h at 37 °C. Then, the glass coverslips were washed twice with PBS at 37 °C, fixed in 4% formaldehyde in PBS for 10 min, permeabilized in 0.25% Triton X-100 solution (Sigma-Aldrich) for 5 min, and washed three times with PBS. AlexaFluor™594 (ThermoFisher Scientific, Carslbad, CA, USA) was applied to label the cytoskeleton, and 4,6-diamidino-2-phenylindole dihydrochloride (DAPI) (ThermoFisher Scientific, Carslbad, CA, USA) was employed to stain the nuclei. Finally, the fluorescently-labeled cells were then observed and photographed via a Leica TCS SP2 confocal microscope (Leica, Wetzlar, Germany). A basal measurement (0 h) was also performed after the placement of the cells into the cell culture. Each solvent eluate concentration was analyzed for three independent experiments and measured in triplicate.

### 2.7. Measurement of Intracellular ROS

Intracellular reactive oxygen species (ROS) levels in those hPDLSCs treated with the different eluates of the tested solvents were quantified by flow cytometry using the general oxidative stress indicator 5-(and-6)-chloromethyl-2′,7′-dichlorodihydrofluorescein diacetate (CM-H2DCFDA) (Molecular Probes, Eugene, OR, USA). In brief, the cells were detached with TrypLE Express dissociation reagent (Thermo Fisher Scientific, Waltham, MA, USA), rinsed twice with DPBS (Gibco, Waltham, MA, USA) and stained with 5 μmol/L CM-H2DCFDA in the dark for 30 min at 37 °C. Then, intracellular fluorescence was quantified using a LSR Fortessa X-20 flow cytometer (Becton Dickinson, Franklin Lakes, NJ, USA) at an excitation of 492 nm and emission of 517 nm. Finally, CM-H2DCFDA positive cells were analyzed with FlowJo software (FlowJo LLC, Ashland, OR, USA). All experimental conditions were analyzed in *n* = 3 experiments.

### 2.8. Statistical Analysis

Graph-Pad Prism v8.1.0 (GraphPad Software, San Diego, CA, USA) was used to perform the statistical analyses. Data compatibility with normal distribution was analyzed and confirmed via a Q-Q plot. Data were analyzed using one-way ANOVA and Tukey’s posthoc test, and significance was considered at *p* < 0.05.

## 3. Results

### 3.1. Trypan Blue Assay

**Figure 1:**
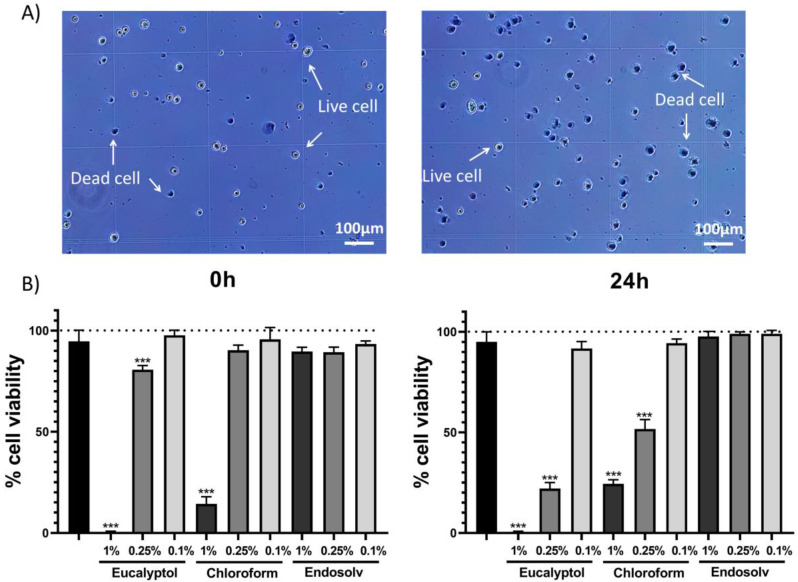
Trypan blue assay results. (**A**) Representative images of the live/dead cell count (scale bar 100 µm). (**B**) Percentage of cell viability at 0 and 24 h for the tested solvent eluates (1%, 0.25%, and 0.1%). *** *p* < 0.001.

Trypan blue assay was performed to determine the alive or dead cells at different time points (0 h and 24 h of culture). After exposure, the hPDLSCs were then placed in a haemocytometer, and the number of viable (unstained) and dead (stained) cells were counted (Figure 1A). The 1% eucalyptol and chloroform-treated cells exhibited significantly lower viability compared to the control group at both time points (*p* < 0.001), whereas the 1% Endosolv group showed similar viability to that of the control group for all concentrations and time points; remarkably, the 0.25% eucalyptol group displayed significantly lower viability compared to the control group at both time points (*p* < 0.001), whereas the 0.25% chloroform-treated cells showed lower viability compared to the control group after 24 h of exposure (*p* < 0.001) (Figure 1B).

### 3.2. IC50 Assay

At 0 h, the IC50 values (i.e., the percentage concentration of each solvent to inhibit 50% of hPDLSC viability) were, after 2, 10, and 20 min of exposure, eucalyptol = 0.19, 0.18, and 0.17%, respectively; chloroform = 0.99, 0.72, and 0.42%, respectively, and Endosolv = 3.36, 1.52, 1.30%, respectively.

**Figure 2:**
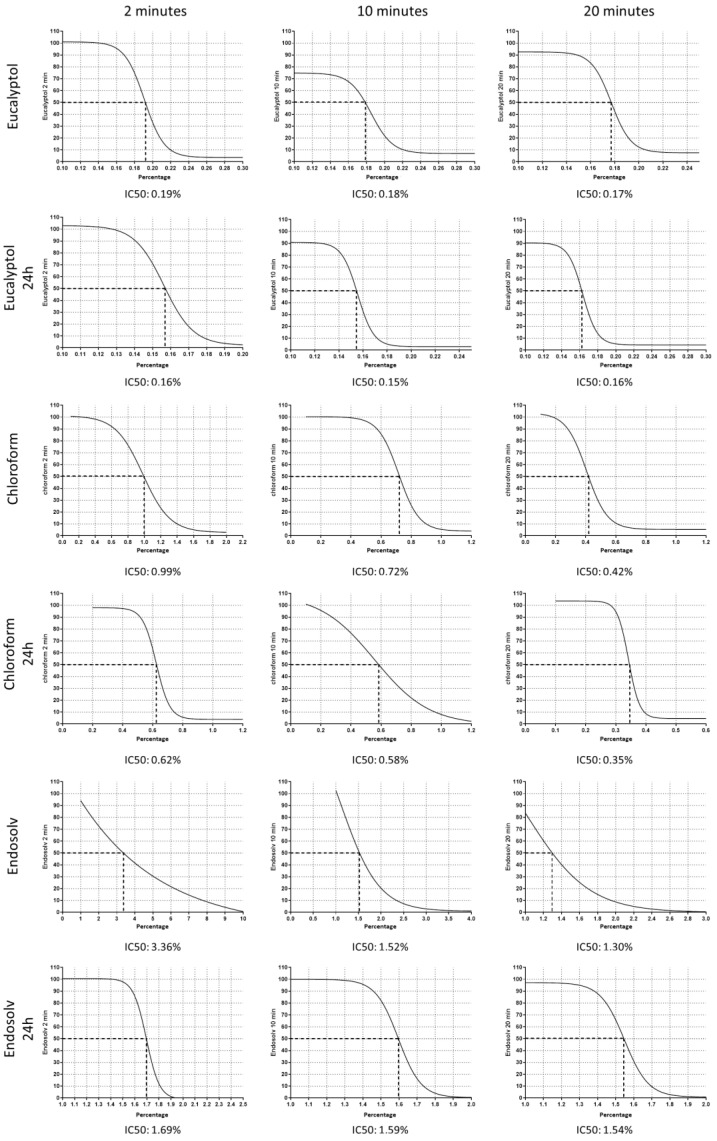
IC50 assay results. The data are illustrated graphically by plotting the percentage of metabolic activity on the *y*-axis and the concentration percentage of each solvent on the *x*-axis. The data are presented at 2, 10, and 20 min at 0 h and after 24 h of culture.

After 24 h of culture, the IC50 values were, after 2, 10, and 20 min of exposure, eucalyptol = 0.16, 0.15, and 0.16%, respectively; chloroform = 0.66, 0.58, and 0.35%, respectively, and Endosolv = 1.69, 1.59, and 1.54%, respectively (Figure 2).

### 3.3. Migration Assay

**Figure 3:**
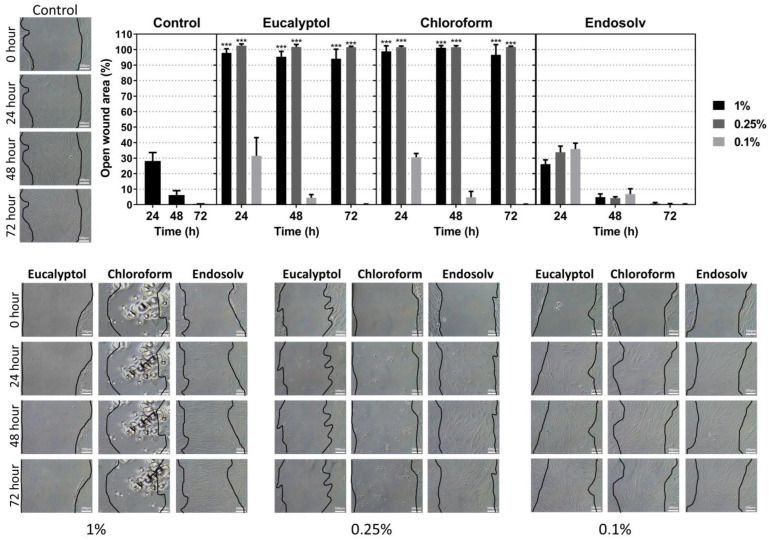
Wound healing assay results. The percentage of open wound area is shown graphically at three different time points (24, 48, and 72 h) for each of the tested solvent eluates (1%, 0.25%, and 0.1%) relative to the percentage of open wound area at 0 h. *** *p* < 0.001. Images of the open wound area are also shown (scale bar 100 µm).

The results of the migration ability of hPDLSCs exposed to the solvent eluates are presented in Figure 3. The Endosolv-treated groups promoted similar wound closure compared with the control. In contrast, the 1% and 0.25% eucalyptol and chloroform-treated cells exhibited a significant decrease in migration ability (*p* < 0.001), corroborating the cell viability assay results.

### 3.4. Cell Cytoskeleton Staining

**Figure 4:**
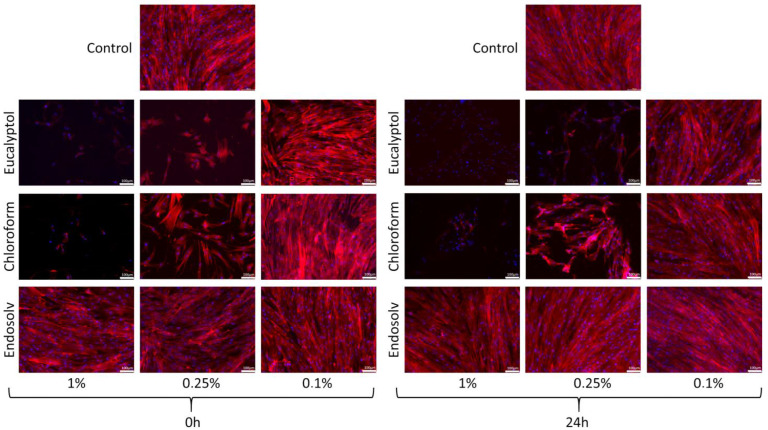
Cell cytoskeleton staining results. Confocal images of the hPDLSCs after treatment with the tested solvents. Blue fluorescence indicates cell nuclei; red fluorescence indicates the actin cytoskeleton. Scale bar = 100 μm.

The Endosolv-treated cells exhibited a flattened and wide-spread cell body with a structured cytoskeleton at all concentrations (1, 0.25, and 0.1%) and both time points (0 h and 24 h after culture), whereas this phenomenon only occurred with 0.1% Eucalyptol and chloroform-treated cells. Conversely, no detectable cells were observed in the 1% eucalyptol and chloroform groups, whereas the cytoskeleton of the 0.25% eucalyptol and chloroform-treated cells was poorly organized and appeared partially disrupted (Figure 4).

### 3.5. Effect of Solvents on Intracellular ROS Production

**Figure 5:**
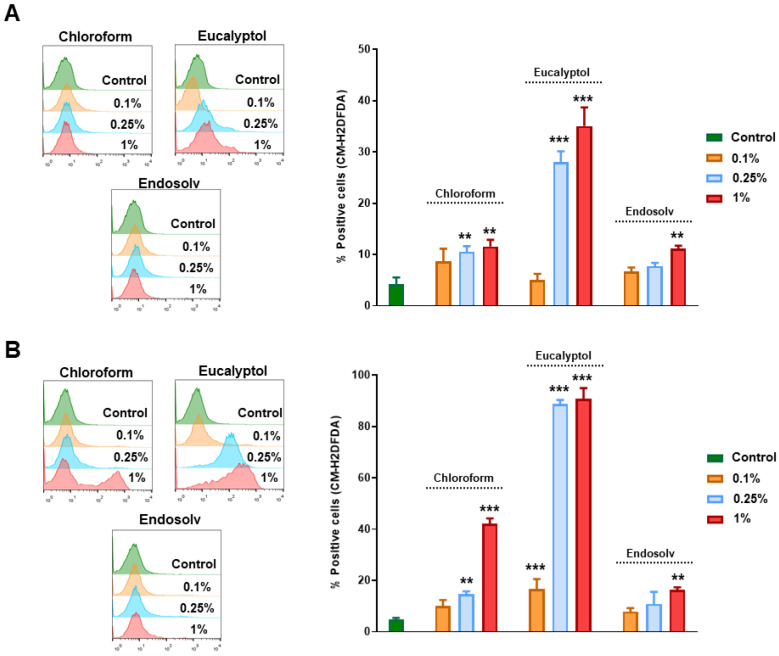
ROS release results on the percentage of CM-H2DCFDA-positive cells after culture with the tested solvent eluates for 0 h (**A**) and 24 h (**B**) when compared to the control group. ** *p* < 0.01, *** *p* < 0.001.

As shown in Figure 5, we investigated the intracellular ROS production on those hPDLSCs treated with several concentrations of the different solvents (0.1%, 0.25%, and 1%) for 0 h and 24 h. At 0 h, the hPDLSCs treated with 0.25% and 1% of chloroform and eucalyptol showed a significant increase in ROS production compared to control cells, mainly with Eucalyptol (** *p* < 0.01, *** *p* < 0.001). On the other hand, Endosolv only increased the ROS levels at the highest concentration (** *p* < 0.01) (Figure 5A). Interestingly, these increased ROS levels were more pronounced when cells were exposed to the more concentrated solvents for 24 h, mainly with eucalyptol (** *p* < 0.01, *** *p* < 0.001) (Figure 5B).

## 4. Discussion

To date, evidence on endodontic solvents centers around their effectiveness in removing gutta-percha and root canal sealers [3]. Nevertheless, a series of studies can be found on other topics, such as those covering their effect on the shear bond strength of restorative materials [22], antibacterial properties [23], their impact on apically extruded debris [24], their influence on electronic apex locator accuracy [25], and their association with postoperative pain [26], among others. However, the biological properties of these materials remain unelucidated. Accordingly, the present study aimed to assess the biological response of hPDLSCs to treatment with endodontic solvents.

For the biological assays, previously characterized hPDLSCs [27] were used as the tested cell population. These cells have demonstrated high long-term survival, self-renewal capability, and the ability to form mineralized bone/cement-like tissue [28,29]. In addition, their use as the target cell population for the in vitro assessment of the biological properties of endodontic materials extends among the literature [30,31]. Regarding sample preparation, three different concentrations of solvent eluates were used (1%, 0.25%, and 0.1%), as performed in previous laboratory studies on other endodontic materials [32]. The justification for the use of different concentrations resides on the fact that, during root canal retreatment, variable proportions of irrigants, solvents, gutta-percha, and sealers can reach the apical portion of the roots and extrude towards the supporting tissues [33,34]. In this way, it is hoped that the clinical response of periapical cellular populations can be better anticipated.

The assessment of cell viability was performed by means of trypan blue assay, as performed in previous studies [14,15,35]. In other studies on the biological properties of endodontic materials, methylthiazole tetrazolium (MTT) colorimetric assays were used as an alternative. It has been previously described that MTT assays are more discriminatory and sensitive in terms of the detection of limited loss of cell viability after exposure to low concentrations of cytotoxic samples. Alternatively, trypan blue assays allow the counting of both dead and viable cells after exposure to higher concentrations of the samples [36]. In other words, trypan blue can be a suitable option to discriminate dead/viable cells in cases where large percentages of cell death are to be expected, such as in the present study.

As an additional measurement of cell viability, a complementary IC50 assay was performed to verify the results of the trypan blue assay, as performed in previous studies [37,38]. In a similar manner, the selection of the remaining biological assays was based on previous studies on the biological properties of endodontic materials [39]. The same occurs with the measurement time points for each of the assays. It should be highlighted that an inherent limitation of these assays is the use of a basal 0 h measurement time point, which does not exactly correspond to 0, but may range from 0 to a few minutes in some cases.

The results from the cell viability assays (trypan blue and IC50 assays) from the present study highlight the cytotoxicity of all the tested dilutions of chloroform and eucalyptol. Endosolv, on the other hand, showed comparable results to those of the control group in the cell viability assays. In accordance with the trypan blue and IC50 assays, we also observed the same pattern in terms of hPDLSC migration and ROS release. Dental stem cells may produce excessive levels of intracellular ROS upon certain circumstances like low nutrient supply, bacterial infection, or low oxygen levels. Excessive levels of ROS can lead to the oxidation of cellular DNA, proteins, lipids, and membranes. Altogether, this may affect cell architecture and integrity [40].

The dose-dependency of the exhibited cytotoxicity was also observed, such that the highest dilutions of the tested solvents exhibited lower cytotoxicity. Clinically, the tested materials are used without diluting them (according to the manufacturer’s instructions). Nevertheless, the concentration of solvent that reaches the periapical tissues may vary depending on the characteristics of the root canal system and irrigation procedure.

These results are in accordance with previous evidence on the biological response of L929 murine fibroblasts toward endodontic solvents [4,41,42]. Altogether, these studies highlight the significant cytotoxicity of eucalyptol, chloroform, limonene, halothane, and turpentine in a time- and dose-dependent manner.

Studies on other animal cell lines have also been previously performed, such as Swiss mice peritoneal macrophages [14], mice lymphoma cells [15], or Chinese hamster ovary cells [34]. Again, these studies reported significantly higher cytotoxicity of eucalyptol and chloroform compared to a control group. Nevertheless, it should be highlighted that previous studies have reported that data from the cytotoxicity screening of human cell lines is more valuable than animal cell lines since human cell lines are more sensitive to these tests [43,44].

To the author’s knowledge, only three studies have been performed on human cell lines, namely human osteoblasts [4,5] and human gingival fibroblasts [45]. The study by Chang & Chou reported the cytotoxicity of halothane on human gingival fibroblast cultures in a concentration- and time-dependent manner, coinciding with a previous study on the same solvent using an L929 cell line [41]. In the study by Gondogan et al., however, both L929 fibroblasts and human osteoblasts were assessed, describing the negative influence of chloroform, orange oil, and eucalyptol on the migration of both cell lines in a dose- and time-dependent manner. However, the authors highlighted that neither of the tested solvents altered the proliferation of human osteoblasts, concluding that these cells seemed more resistant to the tested solvents [4]. These results support the use of human cell lines to test the biological properties of endodontic solvents, as performed in the present study, since the results with animal cell lines may differ.

Lastly, the study by Ferreira et al. assessed the cytotoxicity of three dilutions of tetrachloroethylene, orange oil, chloroform, and methyl ethyl ketone (MEK) on M663 human osteoblastic cells [5]. In addition, the combination of MEK with tetrachloroethylene or orange oil was also assessed. The undiluted solvents exhibited the highest cytotoxicity, coinciding with previous studies and with the present study. Chloroform, however, exhibited high levels of cytotoxicity in all its tested dilutions. Interestingly, the tested mixtures showed no significant differences with the control group for their lowest dilutions. These promising results, however, require future confirmation on alternative cell lines.

Altogether, the evidence highlights the cytotoxicity of chloroform towards several animal and human cell lines. Essential oils, such as eucalyptol or orange oil, which appeared to be biocompatible alternatives, also showed increased cytotoxicity. These solvents have shown high effectiveness in the removal of gutta-percha in root canal retreatment [5,7,46]. However, based on the results of the previously described evidence and from the present study, its limited and cautious use is recommended. Special attention should be paid to the prevention of solvent extrusion, distinctively in cases with wide root canal orifices or root perforations.

On the other hand, Endosolv exhibited the lowest cytotoxicity, comparable to that of the control group. In addition, previous studies have shown its effectiveness in removal or root canal sealers [47,48]. Thus, based on the results of the present in vitro study, the use of Endosolv as a root canal sealer solvent can be recommended from a biological perspective. Nonetheless, future studies should confirm its adequate biological properties in other local cell lines, such as human osteoblasts, cementoblasts, or fibroblasts.

To our knowledge, this is the first study to describe the biological response of hPDLSCs toward endodontic solvents. The reported results, however, should be interpreted with caution due to the laboratory-based nature of the assays. The response from the hPDLSCs was observed in vitro. Clinically, the expressed behavior may vary due to several influencing factors, such as age, hypoxia, or patients’ immune response [49,50,51]. Instead, our results should act as preliminary evidence for the development of future studies on different cell lines, animal models, and/or clinical trials.

## 5. Conclusions

Chloroform and eucalyptol exhibited high cytotoxicity on human periodontal ligament stem cells in vitro, as was shown by cell viability, migration, morphology, and reactive oxygen species release assays. Alternatively, Endosolv showed adequate cytotoxicity levels, which were comparable to those of the control group. The cytotoxicity of the tested endodontic solvents increased in a dose-dependent manner. The limited and cautious use of chloroform and eucalyptol is recommended, avoiding solvent extrusion.

## Figures and Tables

**Table 1:** Studied materials.

Materials	Manufacturer	Composition	Batch Number
Eucalyptol	DF DentaFlux (Madrid, Spain)	Eucalyptol 99% minimum	010921
Endosolv	Septodont (Saint-Maur-des-Fossés, France)	Ethyl acetate (50–100%), Amyl acetate (2.5–10%) and thymol (<1%)	21121
Chloroform	Panreac AppliChem—ITW Reagents (Glenview, IL, USA)	Chloroform 99% minimum	142155370

## Data Availability

The data presented in this study are available on request from the corresponding author.

## References

[1] Nair V., Das S., De Ida A., Das S., Saha N., Chattopadhyay S. (2017). Comparative evaluation of three different rotary instrumentation systems for removal of gutta-percha from root canal during endodontic retreatment: An in vitro study. J. Conserv. Dent..

[2] Yang R., Han Y., Liu Z., Xu Z., Liu H., Wei X. (2021). Comparison of the efficacy of laser-activated and ultrasonic-activated techniques for the removal of tricalcium silicate-based sealers and gutta-percha in root canal retreatment: A microtomography and scanning electron microscopy study. BMC Oral Health.

[3] Dotto L., Sarkis-Onofre R., Bacchi A., Pereira G.K.R. (2021). The use of solvents for gutta-percha dissolution/removal during endodontic retreatments: A scoping review. J. Biomed. Mater. Res. Part B Appl. Biomater..

[4] Gundogan G.I., Durmus S., Ozturk G.C., Kucukyesil N., Acar Y.T., Balaban R., Kig C. (2021). A comparative study of the effects of gutta-percha solvents on human osteoblasts and murine fibroblasts. Aust. Endod. J..

[5] Ferreira I., Grenho L., Gomes P., Braga A.C., Fernandes M.H., Lopes M.A., Pina-Vaz I. (2020). Efficacy and Cytotoxicity of Binary Mixtures as Root Canal Filling Solvents. Materials.

[6] Mushtaq M., Masoodi A., Farooq R., Khan F. (2012). The Dissolving Ability of Different Organic Solvents on Three Different Root Canal Sealers: In Vitro Study. Iran Endod. J..

[7] Martos J., Bassotto A.P.S., González-Rodríguez M.P., Ferrer-Luque C.M. (2011). Dissolving efficacy of eucalyptus and orange oil, xylol and chloroform solvents on different root canal sealers. Int. Endod. J..

[8] Seo B.-M., Miura M., Gronthos S., Bartold P.M., Batouli S., Brahim J., Young M., Robey P.G., Wang C.Y., Shi S. (2004). Investigation of multipotent postnatal stem cells from human periodontal ligament. Lancet.

[9] Bartold P., Gronthos S. (2017). Standardization of Criteria Defining Periodontal Ligament Stem Cells. J. Dent. Res..

[10] Bright R., Hynes K., Gronthos S., Bartold P.M. (2015). Periodontal ligament-derived cells for periodontal regeneration in animal models: A systematic review. J. Periodontal Res..

[11] Schmalz G., Widbiller M., Galler K. (2016). Material Tissue Interaction—From Toxicity to Tissue Regeneration. Oper. Dent..

[12] Sequeira D.B., Seabra C.M., Palma P.J., Cardoso A.L., Peça J., Santos J.M. (2018). Effects of a New Bioceramic Material on Human Apical Papilla Cells. J. Funct. Biomater..

[13] Li X., Pedano M.S., Li S., Sun Z., Jeanneau C., About I., Hauben E., Chen Z., Van Landuyt K., Van Meerbeek B. (2020). Preclinical effectiveness of an experimental tricalcium silicate cement on pulpal repair. Mater. Sci. Eng. C.

[14] Scelza M.F.Z., Oliveira L.R.L., Carvalho F.B., Faria S.C.-R. (2006). In vitro evaluation of macrophage viability after incubation in orange oil, eucalyptol, and chloroform. Oral Surg. Oral Med. Oral Pathol. Oral Radiol. Endodontol..

[15] Ribeiro D.A., Marques M.E.A., Salvador D.M.F. (2006). In vitro cytotoxic and non-genotoxic effects of gutta-percha solvents on mouse lymphoma cells by single cell gel (comet) assay. Braz. Dent. J..

[16] Nagendrababu V., Murray P.E., Ordinola-Zapata R., Peters O.A., Rôças I.N., Siqueira J.F., Priya E., Jayaraman J., Pulikkotil S., Camilleri J. (2021). PRILE 2021 guidelines for reporting laboratory studies in Endodontology: A consensus-based development. Int. Endod. J..

[17] Rodríguez-Lozano F., Collado-González M.D.M., Tomás-Catalá C., Garcia S.L., López S., Sánchez R.E.O., Moraleda J., Murcia L. (2019). GuttaFlow Bioseal promotes spontaneous differentiation of human periodontal ligament stem cells into cementoblast-like cells. Dent. Mater..

[18] Dominici M., Le Blanc K., Mueller I., Slaper-Cortenbach I., Marini F.C., Krause D.S., Deans R.J., Keating A., Prockop D.J., Horwitz E.M. (2006). Minimal criteria for defining multipotent mesenchymal stromal cells. The International Society for Cellular Therapy position statement. Cytotherapy.

[19] Sanz J.L., López-García S., Lozano A., Pecci-Lloret M.P., Llena C., Guerrero-Gironés J., Rodríguez-Lozano F.J., Forner L. (2020). Microstructural composition, ion release, and bioactive potential of new premixed calcium silicate–based endodontic sealers indicated for warm vertical compaction technique. Clin. Oral Investig..

[20] López-García S., Pecci-Lloret M.P., Pecci-Lloret M.R., Guerrero-Gironés J., Rodríguez-Lozano F.J., García-Bernal D. (2021). Topical fluoride varnishes promote several biological responses on human gingival cells. Ann. Anat.—Anat. Anz..

[21] Rodríguez-Lozano F.J., López-García S., García-Bernal D., Tomás-Catalá C.J., Santos J.M., Llena C., Lozano A., Murcia L., Forner L. (2020). Chemical composition and bioactivity potential of the new Endosequence BC Sealer formulation HiFlow. Int. Endod. J..

[22] Ghouchani T.Z., Farhadpour H., Mohammadi N. (2021). Effect of Root Canal Filling Materials and Pretreatment with Solvents on the Shear Bond Strength of Composite Resin with Primary Tooth Dentin. BioMed Res. Int..

[23] Maria R., Dutta S.D., Thete S.G., AlAttas M.H. (2021). Evaluation of Antibacterial Properties of Organic Gutta-percha Solvents and Synthetic Solvents against *Enterococcus faecalis*. J. Int. Soc. Prev. Community Dent..

[24] Çanakçi B.C., Er O., Dincer A. (2015). Do the Sealer Solvents Used Affect Apically Extruded Debris in Retreatment?. J. Endod..

[25] Al-Hadlaq S.M. (2013). Effect of chloroform, orange solvent and eucalyptol on the accuracy of four electronic apex locators. Aust. Endod. J..

[26] Sen O.G., Erdemir A., Canakci B.C. (2021). Effect of solvent use on postoperative pain in root canal retreatment: A randomized, controlled clinical trial. Clin. Oral Investig..

[27] López-García S., Myong-Hyun B., Lozano A., García-Bernal D., Forner L., Llena C., Guerrero-Gironés J., Murcia L., Rodríguez-Lozano F.J. (2021). Cytocompatibility, bioactivity potential, and ion release of three premixed calcium silicate-based sealers. Clin. Oral Investig..

[28] Han J., Menicanin D., Marino V., Ge S., Mrozik K., Gronthos S., Bartold P.M. (2014). Assessment of the regenerative potential of allogeneic periodontal ligament stem cells in a rodent periodontal defect model. J. Periodontal Res..

[29] Menicanin D., Mrozik K., Wada N., Marino V., Shi S., Bartold P., Gronthos S. (2014). Periodontal-Ligament-Derived Stem Cells Exhibit the Capacity for Long-Term Survival, Self-Renewal, and Regeneration of Multiple Tissue Types in Vivo. Stem Cells Dev..

[30] Wang Y., Zhou Y., Jin L., Pang X., Lu Y., Wang Z., Yu Y., Yu J. (2018). Mineral trioxide aggregate enhances the osteogenic capacity of periodontal ligament stem cells via NF-κB and MAPK signaling pathways. J. Cell. Physiol..

[31] Sanz J.L., Guerrero-Gironés J., Pecci-Lloret M.P., Pecci-Lloret M.R., Melo M. (2021). Biological interactions between calcium silicate-based endodontic biomaterials and periodontal ligament stem cells: A systematic review of in vitro studies. Int. Endod. J..

[32] Collado-González M., López-García S., García-Bernal D., Oñate-Sánchez R.E., Tomás-Catalá C.J., Moraleda J.M., Lozano A., Forner L., Rodríguez-Lozano F.J. (2019). Biological effects of acid-eroded MTA Repair HP and ProRoot MTA on human periodontal ligament stem cells. Clin. Oral Investig..

[33] Aminoshariae A., Kulild J.C. (2020). The impact of sealer extrusion on endodontic outcome: A systematic review with meta-analysis. Aust. Endod. J..

[34] Keskin C., Sariyilmaz E., Sariyilmaz O. (2017). Effect of solvents on apically extruded debris and irrigant during root canal retreatment using reciprocating instruments. Int. Endod. J..

[35] Ribeiro D.A., Matsumoto M.A., Marques M.E., Salvadori D.M. (2017). Biocompatibility of gutta-percha solvents using in vitro mammalian test-system. Oral Surg. Oral Med. Oral Pathol. Oral Radiol. Endodontol..

[36] Da Costa A.O., De Assis M.C., De A Marques E., Plotkowski M.C. (1999). Comparative analysis of three methods to assess viability of mammalian cells in culture. Biocell.

[37] Vouzara T., Ziouti F., Economides N., Koulaouzidou E. (2016). Combined and independent cytotoxicity of sodium hypochlorite, ethylenediaminetetraacetic acid and chlorhexidine. Int. Endod. J..

[38] Segura-Egea J.J., Jiménez-Rubio A., Rios-Santos J.V., Velasco-Ortega E., Calvo J.R. (2003). In Vitro Inhibitory Effect of EGTA on Macrophage Adhesion: Endodontic Implications. J. Endod..

[39] Sanz J.L., Soler-Doria A., López-García S., García-Bernal D., Rodríguez-Lozano F.J., Lozano A., Llena C., Forner L., Guerrero-Gironés J., Melo M. (2021). Comparative Biological Properties and Mineralization Potential of 3 Endodontic Materials for Vital Pulp Therapy: Theracal PT, Theracal LC, and Biodentine on Human Dental Pulp Stem Cells. J. Endod..

[40] Zhang J., Lan T., Han X., Xu Y., Liao L., Xie L., Yang B., Tian W., Guo W. (2021). Improvement of ECM-based bioroot regeneration via N-acetylcysteine-induced antioxidative effects. Stem Cell Res. Ther..

[41] Vajrabhaya L.-O., Suwannawong S.K., Kamolroongwarakul R., Pewklieng L. (2004). Cytotoxicity evaluation of gutta-percha solvents: Chloroform and GP-Solvent (limonene). Oral Surg. Oral Med. Oral Pathol. Oral Radiol. Endodontol..

[42] Barbosa S.V., Burkard D.H., Spångberg L.S. (1994). Cytotoxic effects of gutta-percha solvents. J. Endod..

[43] Illeperuma R.P., Park Y.J., Kim J.M., Bae J.Y., Che Z.M., Son H.K., Han M.R., Kim K.-M., Kim J. (2012). Immortalized gingival fibroblasts as a cytotoxicity test model for dental materials. J. Mater. Sci. Mater. Med..

[44] Rodríguez-Lozano F.J., Serrano-Belmonte I., Pérez Calvo J.C., Coronado-Parra M.T., Bernabeu-Esclapez A., Moraleda J.M. (2013). Effects of two low-shrinkage composites on dental stem cells (viability, cell damaged or apoptosis and mesenchymal markers expression). J. Mater. Sci. Mater. Med..

[45] Chang Y.-C., Chou M.-Y. (2001). Cytotoxicity of Halothane on Human Gingival Fibroblast Cultures In Vitro. J. Endod..

[46] Khan F.R., Rehman K., Aman N. (2013). Comparison of Orange Oil and Chloroform as Gutta- Percha Solvents in Endodontic Retreatment. J. Contemp. Dent. Pr..

[47] Tyagi S., Choudhary E., Choudhary A., Chauhan R. (2020). A Comparative Evaluation of Two Commonly Used GP Solvents on Different Epoxy Resin-based Sealers: An In Vitro Study. Int. J. Clin. Pediatr. Dent..

[48] Hwang J.I., Chuang A.H., Sidow S.J., McNally K., Goodin J.L., McPherson J.C. (2015). The Effectiveness of Endodontic Solvents to Remove Endodontic Sealers. Mil. Med..

[49] Leprince J.G., Zeitlin B., Tolar M., Peters O. (2012). Interactions between immune system and mesenchymal stem cells in dental pulp and periapical tissues. Int. Endod. J..

[50] Kellner M., Steindorff M.M., Strempel J.F., Winkel A., Kühnel M.P., Stiesch M. (2014). Differences of isolated dental stem cells dependent on donor age and consequences for autologous tooth replacement. Arch. Oral Biol..

[51] Li L., Zhu Y.-Q., Jiang L., Peng W., Ritchie H.H. (2011). Hypoxia Promotes Mineralization of Human Dental Pulp Cells. J. Endod..

